# Surgical treatment for esophageal neurofibroma: report of two cases and review of literature

**DOI:** 10.1186/s12893-020-00950-1

**Published:** 2021-02-17

**Authors:** Zhedong Zhang, Xun Wang, Zuli Zhou, Jun Wang, Guanchao Jiang

**Affiliations:** Department of Thoracic Surgery, Peking University People’s Hospital, Beijing, 100044 China

**Keywords:** Neurofibroma, Esophagus, Video-assisted thoracoscopic surgery (VATS), Endoscopy, Thoracotomy

## Abstract

**Background:**

Neurofibroma of the esophagus, originated from the nerve sheath cells and fibroblasts of the esophageal submucosal plexus or the intestinal intermuscular plexus, is a very rare mesenchymal tumor. Most of the cases are treated by surgical methods. Due to the technical complexity of video-assisted thoracoscopic surgery (VATS), there are few reports in the literature of VATS for esophageal neurofibroma in recent years.

**Case presentation:**

We report on two rare cases of esophageal neurofibroma, one of which is a 52-year-old male patient diagnosed with a 4.6 × 5.7 cm upper esophageal submucosal tumor in physical examination. He was admitted to our hospital and the tumor was enucleated by VATS combined with intraoperative endoscopy. There were no complications after operation, and the patients was discharged on the 16th postoperative day. The other patient was a 76-year-old man, with the main clinical manifestation of dysphagia for over 1 year, diagnosed with an 8.0 × 6.0 × 8.0 cm giant subepithelial mass in the lower esophagus. As the intraoperative exploration revealed the tumor connected tightly with the wall of the esophagus, this patient treated by transthoracic partial esophagectomy. The patient was discharged on the 14th postoperative day, and no signs of post-operative complication during the 53-month follow-up. The diagnosis of esophageal neurofibroma was based on these patients’ postoperative pathological examination. In the latest follow-up, these two patients had no evidence of long-term postoperative complication and recurrence.

**Conclusion:**

This is the first reported case of 5 cm in diameter esophageal neurofibroma treated by VATS. This technique can be a commendable treatment option for esophageal neurofibroma, and the tumor diameter is not an absolute contraindication for thoracoscopy. To reduce the unnecessary damage, surgical method for complete tumor resection needs to be determined according to preoperative imaging and intraoperative conditions, partial esophagectomy can be performed via thoracotomy or thoracoscopy for removing neurofibroma when necessary.

## Background

Benign tumors of esophagus are relatively rare, and leiomyoma and gastrointestinal stromal tumor account for about 70% of esophageal submucosal tumors, while neurofibroma occurred under the esophageal mucosa is only 0.9% of this type of tumor [1]. Surgical resection remains the main treatment for this disease [2], but for tumors with larger diameter, the application of VATS has been somewhat limited due to the technical complexity and obtaining a complete excision of the tumor. Thus, there are few reports in the literature of VATS for esophageal neurofibroma in recent years [3]. Here, we reported two rare cases of neurofibroma of the esophagus treated by surgical resection, one of which is the first reported of a tumor with larger than 5 cm in maximum diameter treated by VATS combined with intraoperative endoscopy, and to explore the optional surgical option for esophageal neurofibroma.

## Case presentation

### Case 1

**Fig. 1:**
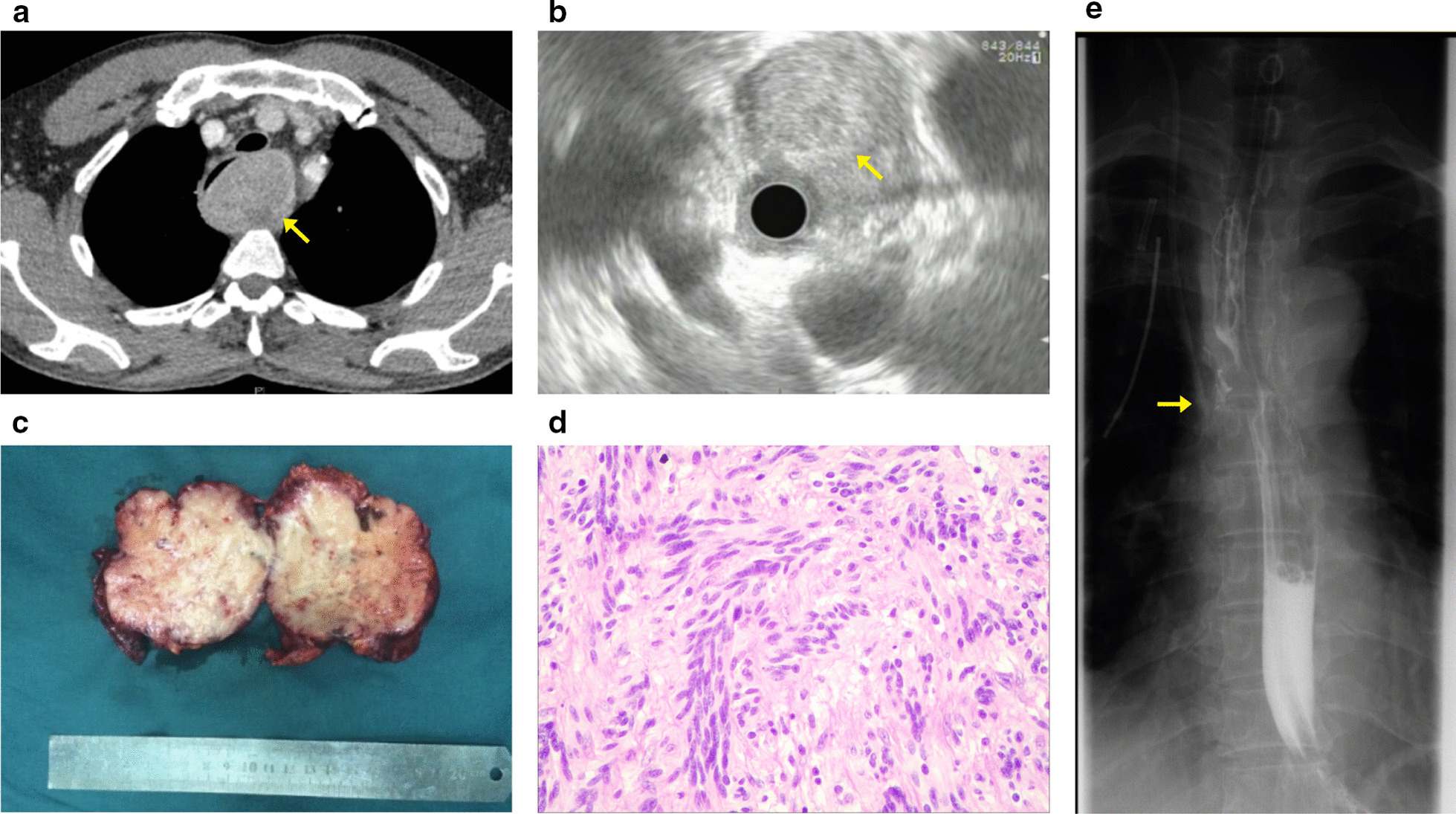
Accompanying clinical data of the case 1. **a** Chest CT revealed the mass at the upper esophagus, with uneven intralesional density. **b** Endosonographic view showed a submucosal mass with smooth boundary. **c** Gross pathological view of removed tumor specimen. **d** Microscopic view of tumor cells. The tumor comprised a mixture of fibrillary collagen and cords of spindle cells with nodular growth and slight cellular heterotopia (H&E stain, original magnification, × 200). **e** Post-operative upper gastroenterography showed no evidence of esophageal leakage and anastomosis narrowing

A 52-year-old man engaged in food processing industry, underwent chest computed tomography (CT) scan for physical examination 10 days ago. The CT revealed a well-circumscribed submucosal tumor measuring 4.6 × 5.7 cm located in the upper esophagus, uneven intralesional density, clear boundary, low-density visible nodular lesion, and progressive heterogeneous enhancement with an enhanced scan (Fig. 1a). He was admitted to our hospital for 10 h of dysphagia. Endoscopic ultrasonography disclosed a submucosal mass with a smooth surface that was located in the midthoracic esophageal region 20 to 26 cm from the incisor teeth, with mixed echo change and clear margins (Fig. 1b). Radiologic and endoscopic findings suggested esophageal leiomyoma or neurogenic tumor. Blood counts, biochemistry values, tumor markers including cytokeratin-19-fragment (CYFRA21-1), carcinoembryonic antigen (CEA) and squamous cell carcinoma antigen (SCC) and cervical-abdominal ultrasound were unremarkable.

After preoperative preparation, VATS was performed under general anesthesia. The patient was placed in the left lateral decubitus position. A small incision (1 cm) was first created at the right sixth intercostal space on the posterior axillary line. Exploration revealed that the tumor was located above the azygos vein, with a diameter of about 5 cm, upwards beyond the top of the pleura, and the trachea was compressed and displaced. Two additional thoracic ports (4 cm and 1 cm) were made on the midaxillary line at the third intercostal spaces and subscapular angular line at the sixth intercostal spaces as the operation hole and for lung retraction, respectively. After the upper esophagus was freed from the posterior mediastinal space, using the intraoperative gastroscopy to reconfirm the tumor's location that is located in the left posterior wall of the esophagus, the esophageal muscle layer was opened along the surface of the tumor. Due to the firm adhesions of the tumor to the esophageal mucosa, the upper part of the esophagus mucosa was damaged during the separation of the tumor. After the tumor was completely peeled off, the damaged part of the esophagus was closed longitudinally in two layers: using interrupted absorbable 4-0 and silk 2-0 sutures for the mucosal and submucosal layer, and the muscular layer, respectively. A 28 Fr chest tube was placed in the esophageal bed and inserted in the sixth intercostal space along the posterior axillary line. The operation approximately took 300 min to complete and estimated intraoperative blood loss was 250 mL. The resected tumor was hard in elasticity, it measured 6.5 × 4.0 × 4.5 cm, and the cut surface was whitish without obvious necrosis (Fig. 1c). The origin of the tumor from the esophageal submucosa and muscularis. Histologic examination demonstrated a large spherical nodular tumor and comprised mixed fibrillary collagen sheets and cords of spindle cells with nodular growth and slight cellular heterotopia. No signs of alternating hypercellular or hypocellular areas were observed. Immunohistochemical staining results were S-100 ( +), SOX10 ( +), Ki-67 (10% +), CD34 (−), αSMA (−), CK(−), EMA(−), CD117(−), DOG1(−), SMA(−), CD34(−) and desmin (−) (Fig. 1d). The above pathologic and immunohistochemical characteristics were consistent with the neurofibroma. The upper gastroenterography was performed on the 14th postoperative day, and it suggested no evidence of esophageal leakage and narrowing (Fig. 1e). There were no complications after operation, and the patient was discharged on the 16th postoperative day. No evidence of long-term postoperative complication and recurrence were observed at the 53-month follow-up.

### Case 2

**Fig. 2:**
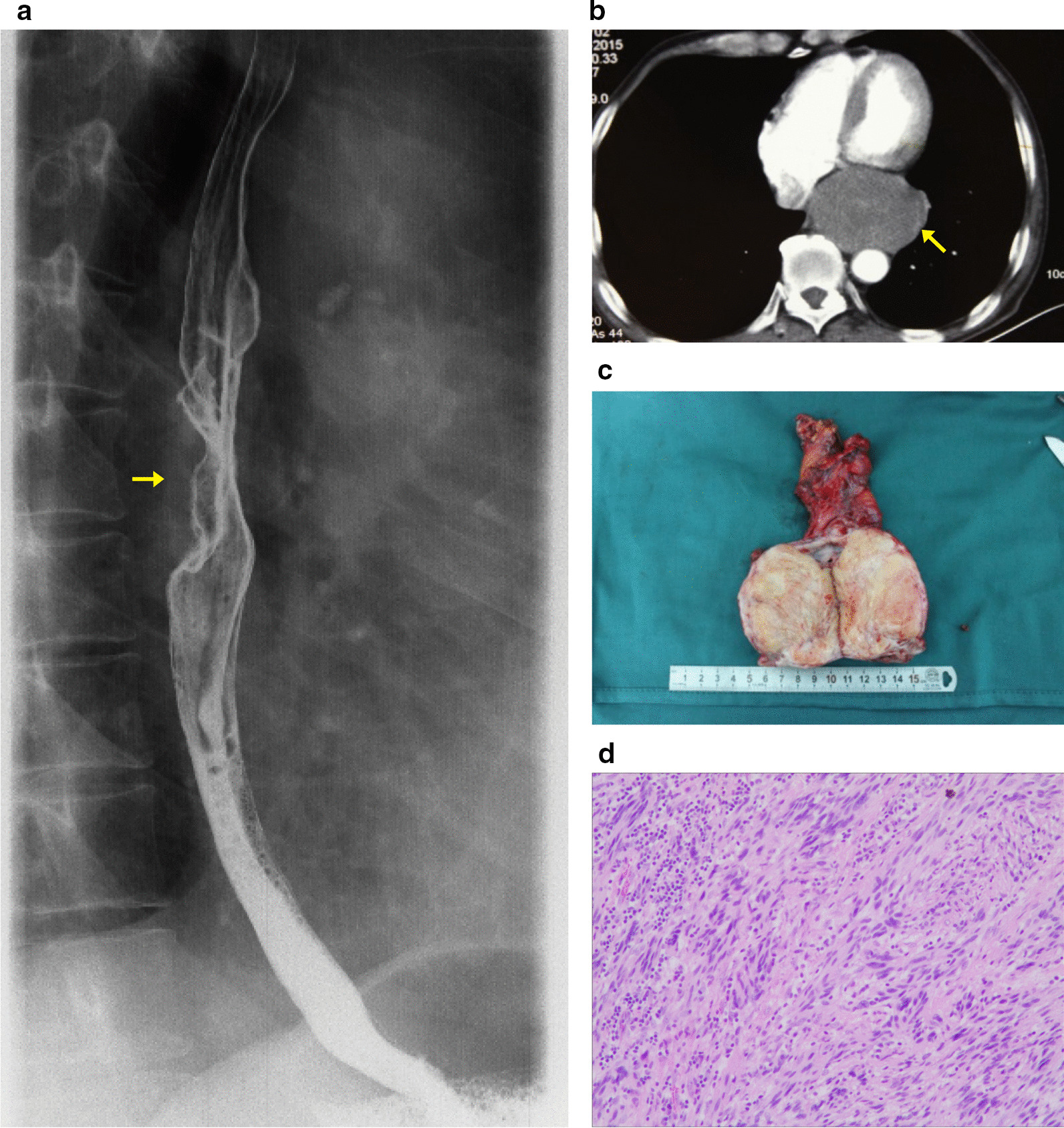
Accompanying clinical data of the case 2. **a** Barium swallow showed a large intraluminal mass of the lower esophagus. **b** The enhanced chest CT revealed a soft tissue shadow of 7.5 cm × 5.4 cm in the lower esophagus, unclear boundary with esophagus. **c** Gross pathological appearance of the tumor. **d** Histologic appearance of the tumor. The tumor comprised a mixture of fibrillary collagen and cords of spindle cells. No signs of cellular atypia, areas of necrosis or mitotic activity were observed (H&E stain, original magnification, × 200)

A 76-year-old male farmer was admitted to our hospital because of dysphagia for over 1 year. Esophagoscopy found that a giant subepithelial mass in the lower esophagus, extending 35 cm from the incisors, with smooth surface. The lumen of the esophagus was stenosed, thus the endoscopy could not pass. A barium swallow showed a large intraluminal mass of the lower esophagus and no significant mucosal damage (Fig. 2a). The enhanced chest CT revealed a soft tissue shadow of 7.5 × 5.4 cm in the lower esophagus, with eccentric expansive growth and unclear margins. The esophagus was compressed and shifted to the left with lumen narrowing. Patchy enhancement could be observed in the arterial phase (Fig. 2b).

With unremarkable preoperative tests and examinations, based on the obscure boundary of tumor with esophagus in preoperative imaging, patient underwent thoracotomy to excise the tumor. A 14Fr duodenal tube was introduced into the patient’s duodenum through the nasal cavity prior to surgery. A 6 cm in diameter thoracotomy was created at the lateral seventh intercostal space, with the patient in the right lateral decubitus position. Exploration showed that a giant tumor, about 8.0 × 6.0 × 8.0 cm in size had filled up the inferior triangle of the esophagus. When the mediastinal pleura was incised and the tumor was free from the surrounding descending aorta and pericardium, we found that the tumor connected tightly with the wall of the esophagus which was consistent with preoperative imaging and could not be dissected completely. Thus, partial esophagectomy and gastroesophageal intrathoracic anastomosis were performed. Firstly, retracting the duodenal tube to the upper part of the esophagus. Secondly, the stomach was cut off with a straight-line suture cutter under the cardia, and the esophagus was transected upward to about 5 cm above the tumor. Thirdly, using linear cutter stapler on lesser curvatures of the stomach to make a gastric conduit. Finally, we reconstructed the esophagus using the gastric conduit. A circle cutter stapler was used for mid-thoracic esophagus and gastric conduit anastomosis through the posterior mediastinum. The anastomosis was reinforced with 3–0 silk. The duodenal nutrition tube was indwelled accessing the esophageal-gastric anastomosis into duodenum under direct vision and a 28 Fr chest tube was routinely placed under the gastroesophageal anastomosis. The operation time was 320 min and estimated intraoperative bleeding was 400 mL.

The cross-sectional specimens show that the tumor is located in the esophageal muscle layer and has invaded the esophageal mucosa (Fig. 2c). Paraffin pathology indicated that there were spindle cells in the submucosa and muscular layer of the esophagus, and a mixture of fibrillary collagen and cords of spindle cells were seen in the tumor. There was no cellular atypia or areas of necrosis, and no mitotic activity (Fig. 2d). Immunohistochemical staining: S-100 ( +), SOX10( +), SMA (Focal area +), desmin(−), CD117(−), Dog-1(−), CD34(−), Ki-67(10% +). The above morphologic and immunohistochemical characteristics were consistent with the diagnosis of neurofibroma. No obvious postoperative complications, the chest tube was removed at the 4th day after surgery, and the patient was discharged on the 14th postoperative day. The patient’s dysphagia resolved after removal of the tumor, and no signs of post-operative complication during the 53-month follow-up.

## Discussion and conclusions

Esophageal neurofibroma is a rare benign submucosal tumor of the esophagus, and the growth rate of the tumor is relatively slow. Booka et al. [4] reviewed 18 cases of localized esophageal neurofibromas which reported in the past. The results showed that the average age of the patients was 53.6 years (26–75 years), and there was no gender predilection for this disease (male:female 8:10). One third of patients had dysphagia symptoms. The location of tumors was primary in the mid to upper part of the thoracic esophagus (83%), and the average diameter was about 6.2 cm (0.5–22.5 cm).

Due to its rarity and non-specific clinical manifestations, this tumor is challenging for preoperative diagnosis, and is easily misdiagnosed as esophageal leiomyoma, gastrointestinal stromal tumor, or even tumors of posterior mediastinal origin. In addition, 25% of patients with Von Recklinghausen’s disease may have gastrointestinal involvement, accompanied by peripheral nerve or (and) cranial nerve multiple neurofibroma and skin pigment spots [5]. Therefore, the reliable diagnostic method for esophageal neurofibroma is still pathological examination. Endoscopic ultrasound (EUS) guided fine-needle aspiration (FNA) allows for biopsy and cytological analysis to help make treatment decisions. Its pathological manifestations are spindle-shaped tumor cells, which may have collagen fibers, and immunohistochemistry can help identify neurogenic or myogenic tumors. In our case, the tumor comprised of fibrillary collagen and spindle cells organized in whorls, and it was positive for S-100 but negative for c-Kit, CD-34, SMA and Desmin.

Up to now, many literatures have reported that surgery is still the main treatment for esophageal neurofibroma. Booka et al. [4] summarized 18 cases in the literature, 4 patients received partial esophagectomy with an average diameter of about 8.5 cm (6–11 cm), and 10 patients received tumor enucleation with an average diameter of about 6.2 cm (0.7–22.5 cm). Therefore, the tumor size was not the only defining factor for the surgical strategy.

Most studies reported that the surgical treatments for esophageal neurofibroma had been performed through thoracotomy or laparotomy. Nishikawa et al. [3] first reported a case with a 3.3 cm in maximal diameter esophageal neurofibroma received enucleation of by VATS. In our study, it is the first reported case treated by VATS combined with intraoperative endoscopy. The esophageal neurofibroma was larger than 5 cm in diameter. Even though, we found that the adhesions between the tumor and esophageal mucosa was resectable by VATS procedure, the intraoperative endoscopy could help to clarify the location of the tumor and confirm whether there was mucosal damage after removing the tumor. This case might demonstrate that VATS combined with intraoperative endoscopy could be considered as an alternative therapeutic option for the management of esophageal neurofibroma.

However, according to these two cases, the best mode of surgery for complete tumor resection still needs to be determined according to preoperative imaging and intraoperative conditions, such as the tumor size, location and boundary. When the tumor is huge and severely adhered to the esophageal mucosa, mucosal tears may occur during the peeling process, intraoperative endoscopy can help to identify the damaged area. Thoracoscopic repair of the esophageal mucosa mostly uses the method of intermittent suture of the esophageal mucosa and muscle layer. For tumors with difficult dissection or large mucosal defects, it is necessary to perform partial thoracic esophagectomy and esophageal reconstruction to ensure complete tumor resection and avoid postoperative esophageal fistula and other complications.

Due to the rarity esophageal neurofibroma, there are few reports of long-term follow-up. Although neurofibroma of esophagus is a benign disease, a long-term clinical follow-up about anastomotic postoperative complications and organ function after surgery is required.

Esophageal neurofibroma is an extremely rare tumor which is difficult to diagnose preoperatively. VATS combined with intraoperative endoscopy as a viable option for the treatment of esophageal neurofibroma. It is necessary to select the best operation mode according to the preoperative radiological examination and intraoperative exploration. This is the first case of 5 cm in diameter esophageal neurofibroma treated by VATS combined with intraoperative endoscopy.

## Data Availability

Not applicable.

## Abbreviations

VATS: Video-assisted thoracoscopic surgery
CT: Computed tomography
CYFRA21-1: Cytokeratin-19-fragment
CEA: Carcinoembryonic antigen
SCC: Squamous cell carcinoma antigen
EUS-FNA: Endoscopic ultrasound guided fine-needle aspiration
